# IDBGL: A unique image dataset of black gram (Vigna mungo) leaves for disease detection and classification

**DOI:** 10.1016/j.dib.2025.111347

**Published:** 2025-01-29

**Authors:** Md. Mehedi Hasan Shoib, Shahnewaz Saeem, Afia Benta Aziz Tonima, Mayen Uddin Mojumdar

**Affiliations:** Multidisciplinary Action Research Lab,Department of Computer Science and Engineering, Daffodil International University, Daffodil Smart City, Birulia, Dhaka 1216, Bangladesh

**Keywords:** Vigna mungo, Deep learning, Machine learning, Disease detection

## Abstract

Black gram (Vigna mungo) is considered one of the most important pulse crops cultivated in Bangladesh because it is a vital source of nutrition and a potential source for raising a good income. It is one of those plants where most leaves are affected by diseases. We observed that most of the leaves were diseased in the fields, and we had difficulty collecting healthy samples. The crop is affected by different diseases attacking leaf tissues, causing heavy yield loss. We can apply deep learning models to recognize diseases in their early stages for timely interference. Diseases could be detected with the automation process, from which much enhancement in the management and yield of black gram crops is possible. Our purpose is to create a unique dataset of Bangladesh's Black Gram (Vigna mungo) to help global researchers build a deep learning-automated system for the early detection and classification of Black Gram leaf diseases that will assist farmers and create more awareness among different agricultural stakeholders. The original dataset of 4,038 images was collected from the Sirajganj and Solonga regions in Bangladesh. The dataset has five different classes: Healthy, Cercospora Leaf Spot, Insect, Leaf Crinkle, and Yellow Mosaic. This dataset will help researchers improve disease detection in Black Grams by developing effective computational models and applying advanced machine learning techniques.

Specifications Table

**Table:** 

Subject	Computer Sciences, Agriculture Sciences.
Specific subject area	Computer Vision, Image Processing, Disease Classification, Machine Learning.
Type of data	The images are in JPEG images (total 4,038 images), with the resolution of 3000 × 4000-pixel dimensions.
Data collection	This dataset consists total of 4,038 Black Gram images, including both Healthy and diseased Leaf. Diseased leaves are categorized into 4 classes: Cercospora Leaf Spot, Insect, Leaf Crinkle, and Yellow Mosaic. The images were captured using: (i) OnePlus 8T, (ii) Samsung A05, (iii) Samsung A15, (iv) Samsung F23, (v) Samsung M33, (vi) Realme Narzo 50, (vii) Realme 5i.
Data source location	Data was collected from the following locations in Bangladesh: 1. Black Gram field Sirajganj Sadar, Sirajganj (Latitude: 24°34′40.4"N, Longitude: 89°38′11.7"E) 2. Black Gram field in Solonga, Sirajganj (Latitude: 24°25′08.7"N, Longitude: 89°30′23.5"E).
Data accessibility	Repository name: Mendeley Data Data identification number: 10.17632/z55yrbmn2d.3 Direct URL to data: https://data.mendeley.com/datasets/z55yrbmn2d/3

## 1 Value of the Data

• This is a dataset dedicated only to black gram (Vigna mungo) leaf diseases, containing valuable resources for both agriculture and machine learning researchers. The classes are well-defined: healthy, Cercospora leaf spot, insect, leaf crinkle, and yellow mosaic. These are diversified sets of images labeled to create and further evaluate the models for automated disease detection.
• The insect disease is rare in the black gram (Vigna mungo) leaf, which class is included in this dataset. This class can help researchers train Machine Learning and deep learning models for rare cases, improving robustness.
• This dataset provides high-quality labeled data, making it possible to develop automated systems for early disease detection [1]. Early disease detection can reduce crop losses and increase the yield and management of black gram crops.
• This dataset enables the creation of precision agricultural technologies, which assist farmers in making timely decisions to control diseases effectively. That can contribute to sustainable farming by minimizing dependency on chemical treatments and maximizing resource utilization.
• This dataset enables collaboration between agricultural sciences and computer vision to tackle crop health management challenges [2].

## 2 Background

In Bangladesh's economy, agriculture plays a vital role. Black Gram (Vigna mungo) is one of the most important pulse crops cultivated in Bangladesh because it is an essential nutritional source and a potential source for raising a good income. But it is one of those plants where most leaves are afflicted by disease. From our observation in the field, we were surprised to see that most of the leaves are unhealthy. The viral infections might lead to reduced agricultural yields, resulting in a substantial economic loss [3]. So, early detection of disease by automated disease detection using machine learning and deep learning models can help farmers give enough to take the necessary steps to reduce the chance of disease [4]. That will minimize crop loss, and the yield will also be good. This dataset of 4,038 images displays several stages of black gram leaf disease, such as healthy, Cercospora leaf spot, insect, leaf crinkle, and yellow mosaic. Each image illustrates the symptoms of black gram leaf disease [5]. This dataset of Black Gram (Vigna mungo) is a valuable resource for researchers in the field of precision agriculture. Researchers can use this dataset to develop automated disease detection systems using machine learning and deep learning models [6]. Finally, this dataset promotes progress in ecological agricultural methods, supporting robustness in black gram production systems worldwide.

## 3 Data Description

The dataset consists of 4,038 high-resolution images, collected from the Sirajganj and Solonga region in Bangladesh. We classified our Black Gram images into a total of five classes. Each Class represents a specific type of leaf. Table 1 shows a full breakdown of the dataset's classes, including the number of original and augmented images.

**Table 1:** Statistics of the black gram dataset.

Class Name	Number of Images
Healthy	545
Cercospora Leaf Spot	598
Leaf Crinkle	806
Insect	408
Yellow Mosaic	1681
**Total**	4038

Five classes of this Black Gram dataset: Healthy, Cercospora Leaf Spot, Insect, Leaf Crinkle, and Yellow Mosaic. This dataset consists of 4,038 images gathered from various sources to simulate real-world situations. We labeled each class with high-quality images to ensure accurate classification. Table 2 highlights the dataset's features, such as the number of images per class, brief descriptions, and sample images for each category.

**Table 2:** Dataset summary of black gram.

Class	Description	Total Images	Sample Images
**Healthy Leaf**	Healthy leaves are vital for photosynthesis and nutrition distribution, are high in vitamins, and help prevent du [3].	545	
**Cercospora leaf spot**	Cercospora leaf spot is caused by the fungus Cercospora canescens that affects black gram and green gram plants [7].	598	
**Insect**	About 250 insects have been recorded feeding on pulse crops. Of these, about one dozen insects including pod borers, stem borers, leaf miners, foliage caterpillars, cutworms, jassids, aphids, and whiteflies are most important [8].	408	
**Leaf crinkle**	Leaf crinkle in black gram, also known as urdbean leaf crinkle disease (ULCD), is a viral disease that causes severe leaf damage and significant yield losses [9].	806	
**Yellow mosaic**	Yellow mosaic disease (YMD) is a serious disease that affects black gram (Vigna mungo L.) and other grain legumes [10]. It's caused by a single-stranded DNA begomovirus [11].	1681	

We compared our dataset to evaluate the effectiveness and uniqueness against closely related datasets from the work of S. Talasila, et al. [3]. Table 3 showcases a detailed comparison of the datasets. We have highlighted the number of images specific to classes. Our dataset contains 4,038 images from 5 classes, namely Healthy, Cercospora Leaf Spot, Insect, Leaf Crinkle, and Yellow Mosaic, both Healthy and Diseased. The other datasets have fewer categories and dataset [3] has some other different categories and a smaller number of images. The closest dataset [3] that can be compared with ours is given in Table 3.

**Table 3:** Comparison with available datasets of back gram.

SL	Classes	Number of Images	
		Our Dataset	S. Talasila et al., [3]
1	Healthy	✔ (545)	✔ (220)
2	Cercospora Leaf Spot	✔ (598)	**X**
3	Leaf Crinkle	✔ (806)	✔ (150)
4	Insect	✔ (408)	**X**
5	Yellow Mosaic	✔ (1681)	✔ (220)

Our data was taken during October and November of 2024. Pictures were taken at a variety of times of day over several days. Several locations allowed a variety of sorts to be captured. Details are given in Table 4.

**Table 4:** Collection details of black gram dataset.

Black Gram Class Name	Weather	Date	Time	Temperature	Camera Device	Location
Healthy	Sunny	15 October 2024	Noon	30°C	Samsung A05 (45%), Samsung A15 (25%), Realme Narzo 50 (30%)	Sirajganj, Solonga
Cercospora Leaf Spot	Sunny	15 October 2024	Morning	26°C	Realme Narzo 50 (50%), Samsung M33 (25%), Samsung F23 (25%)	Sirajganj
Insect	Windy	16 October 2024	Afternoon	27°C	OnePlus 8T (100%)	Sirajganj
Leaf Crinkle	Cloudy	17 October 2024	Morning	26°C	OnePlus 8T (80%) and Samsung A15 (20%)	Sirajganj, Solonga
Yellow Mosaic	Sunny	24 October 2024	Noon	29°C	Samsung A05 (60%) and Samsung A15 (40%)	Sirajganj, Solonga
Leaf Crinkle	Windy	30 October 2024	Afternoon	28°C	Samsung F23 (40%), Samsung A15 (30%), Realme 5i (30%)	Sirajganj
Yellow Mosaic	Cloudy	01 November 2024	Afternoon	27°C	OnePlus 8T (60%), Realme 5i (25%), Samsung M33 (15%)	Sirajganj

## 4 Experimental Design, Materials and Methods

### 4.1 Experimental design

Images of this dataset of black gram (Vigna mungo) leaves were collected from fields in Sirajganj and Solonga, Bangladesh, during the harvesting season from September 2024 to November 2024. Using multiple smartphone cameras, we captured high-quality images of leaves directly from the plants under natural light conditions. Our goal was to include a variety of leaf conditions, from healthy leaves to those affected by diseases like Cercospora Leaf Spot, Insect Damage, Leaf Crinkle, and Yellow Mosaic.

**Fig. 1:**
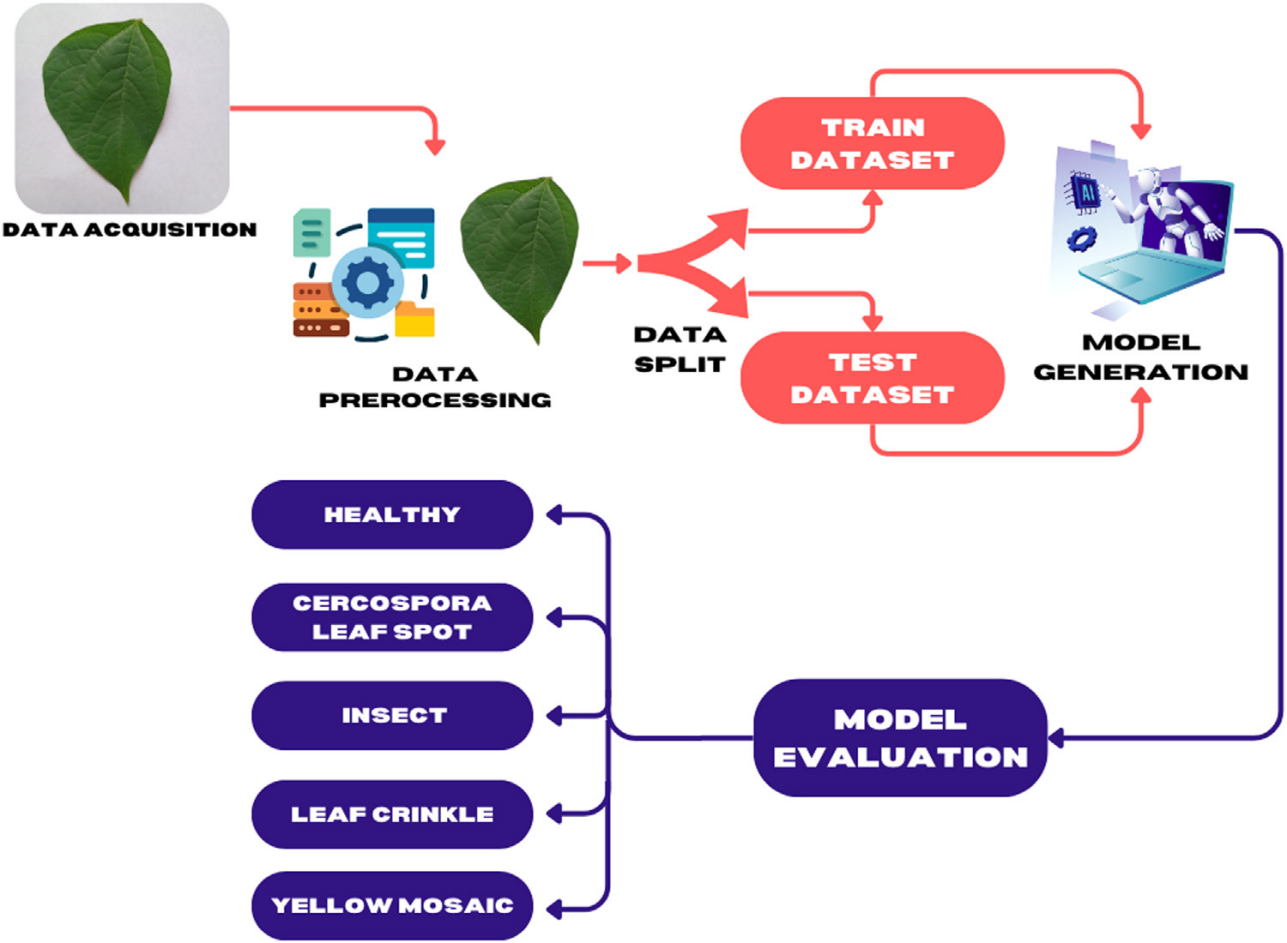
The method by which the diseases of Black Gram leaf are evaluated.

Once the images were collected, we prepared them for analysis by resizing them to a uniform size, cleaning them to remove background noise, replacing them with white backgrounds, and normalizing them with 3000 × 4000 pixel size for better processing. We then split the dataset into two parts: 80% for training and 20% for validation, ensuring a balanced approach for machine learning and deep learning model development. Then we classify images into different classes for the dataset, by applying machine learning model in Fig. 1.

Fig. 1 illustrates the process of building a machine learning-based system for classifying images into ‘Healthy’, ‘Cercospora Leaf Spot’, ‘Insect, Leaf Crinkle’, and ‘Yellow Mosaic’ classes of black gram (Vigna mungo) leaf diseases dataset. It starts with data acquisition, where images of black gram leaves are collected from the field, representing five categories: Healthy, Cercospora Leaf Spot, Insect Damage, Leaf Crinkle, and Yellow Mosaic. Next, the images are cleaned, resized, and normalized during data pre-processing to ensure they are ready for analysis. [12] The dataset is then divided into two parts: a training dataset, which is used to train the machine learning model, and a test dataset, which is used to evaluate the model's performance. A deep learning pre-trained model (such as VGG16) is trained in the model generation step to identify and classify the different Leaves [13]. Finally, the model evaluation phase measures how accurately the system can classify images using accuracy, precision, and recall metrics. By that, the images are classified into these five classes. This workflow shows how a machine learning system can help to classify images into different classes.

### 4.2 Method

We illustrate the pre-processing stages of our Black Gram dataset. We also illustrate augmenting the Black Gram disease dataset for machine learning and deep learning data analysis. Initially, we start the process by acquiring a comprehensive dataset containing images of Black Gram (Vigna mungo affected by several diseases. Then, we conduct particular data cleaning techniques to remove inappropriate or inaccurate data and also ensure the quality of the dataset. After that, we continue with the data processing, which includes standardizing the resolution of images, labeling every image with relevant disease information, and employing advanced segmentation techniques for in-depth analysis [14].

**Fig. 2:**
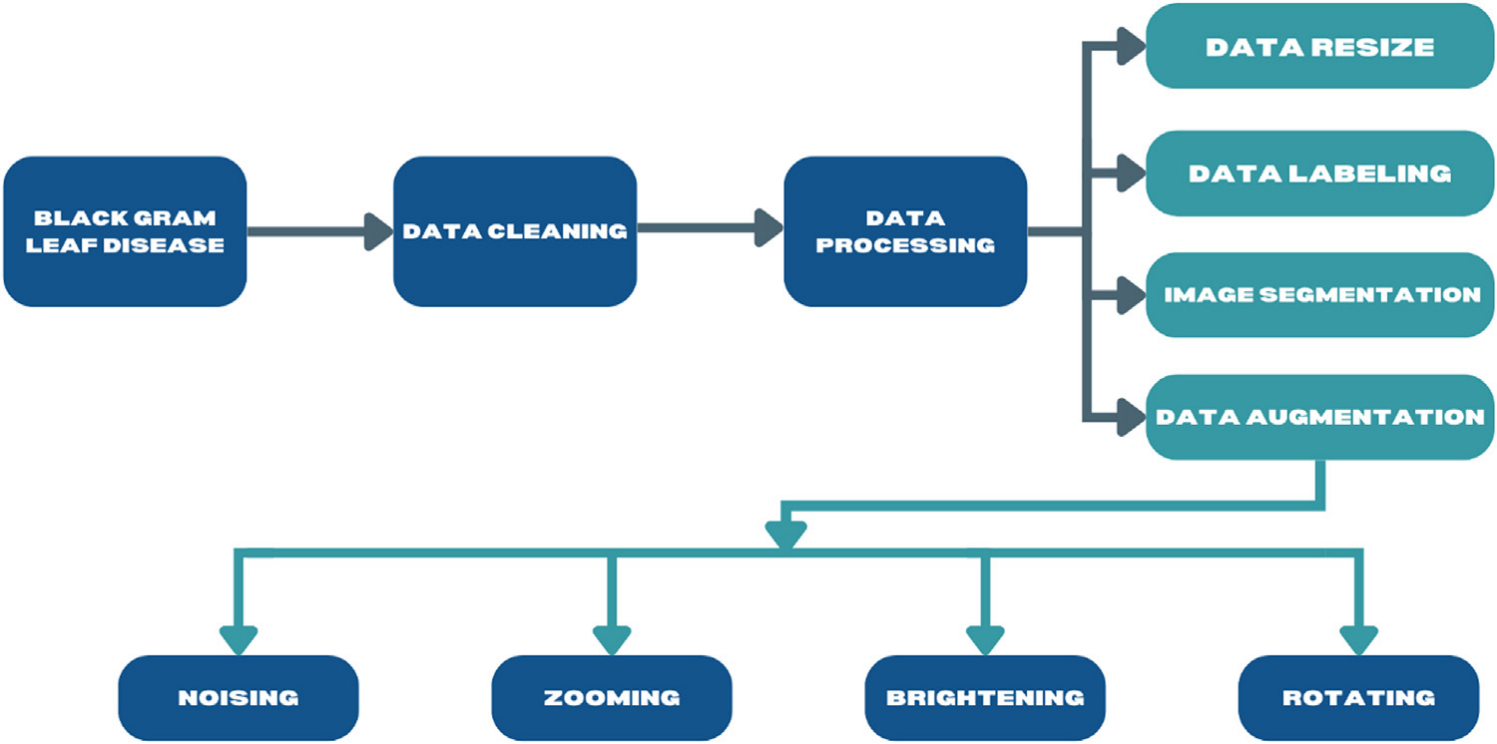
The pre-processing and augmentation stages of the Black Gram disease dataset.

Additionally, we employ data augmentation techniques only for deep learning model validation, such as horizontal rotation, zooming, noising, and brightness, to improve the diversity and resilience of datasets [15]. From initial data cleaning to final data augmentation, the methodical procedure described in Fig. 2 ensures efficient preparation for additional analysis, especially in machine learning or data science applications aimed at detecting and classifying diseases in Black Gram (Vigna mungo).

### 4.3 Data annotation protocol

Annotation involved one highly qualified agronomic, ABDUL MANNAN MOJUMDAR, with vast knowledge of the diagnosis of plant disease and categorization. He served as an additional director in Bangladesh's Department of Agricultural Extension. Actions taken included:

1. Initial Screening: Each image was reviewed to ensure clarity and whether it represented the details of the disease well. Unclear or poor-quality images were excluded from the study.
2. Class Assignment: Based on visible signs, such as discoloration, necrosis, and other forms of deformation, images were then classified into two classes: Healthy and Insect Hole Spot.
3. Verification: The annotations were examined to ensure correctness and consistency across the dataset following the first labeling.

This systematic and expert-processed annotation methodology ensures quality, making the dataset particularly very valuable in machine learning-based disease detection and classification activities [16].

## Limitations

The dataset has some limitations, such as the samples were collected against a plain background, which ensures consistency during data collection and minimizes noise for model training, it may reduce the adaptability of systems trained on this dataset to real-world conditions. Future versions will include samples from diverse, natural environments with varied lighting and textures to improve real-world applicability. Augmenting or synthesizing additional variations could also help address this issue. Also, limitations like variability in natural conditions and being limited to Sirajganj and Solonga, which may affect generalization. While augmented, the original 4,038 images might still be small for complex models. Future work can address these issues to improve its value.

## Ethics Statement

We confirm that we have followed all ethical guidelines. This study did not involve animal experiments or data collected from social media platforms. The data used in this research were collected responsibly from black gram (Vigna mungo) fields, ensuring that no harm was caused to the environment or any individuals during the process.

## CRediT Author Statement

**Md. Mehedi Hasan Shoib:** conceptualization, methodology, writing original draft, data curation. **Shahnewaz Saeem:** Writing, Visualization **Afia Benta Aziz Tonima:** Data curation, Methodology, **Mayen Uddin Mojumdar:** Supervision, formal analysis, writing – review & editing.

## Data Availability

Mendeley DataImage Dataset for Disease Detection in Black Gram (Vigna mungo) Leaves: A Resource for Machine Learning Research (Original data).
